# An overview of multi-omics technologies in rheumatoid arthritis: applications in biomarker and pathway discovery

**DOI:** 10.3389/fimmu.2024.1381272

**Published:** 2024-07-30

**Authors:** Xiangjin Gong, Lanqian Su, Jinbang Huang, Jie Liu, Qinglai Wang, Xiufang Luo, Guanhu Yang, Hao Chi

**Affiliations:** ^1^ Department of Sports Rehabilitation, Southwest Medical University, Luzhou, China; ^2^ Clinical Medical College, Southwest Medical University, Luzhou, China; ^3^ Department of Geriatric, Dazhou Central Hospital, Dazhou, China; ^4^ Orthopedics and Traumatology Department of TCM, Wenzhou TCM Hospital of Zhejiang Chinese Medical University, Wenzhou, China; ^5^ Department of Specialty Medicine, Ohio University, Athens, OH, United States

**Keywords:** rheumatoid arthritis, multi-omics integrative analysis, autoimmune diseases, metabolism, metabolic pathways, dysregulation, therapeutic targets, systems immunology

## Abstract

Rheumatoid arthritis (RA) is a chronic inflammatory autoimmune disease with a complex pathological mechanism involving autoimmune response, local inflammation and bone destruction. Metabolic pathways play an important role in immune-related diseases and their immune responses. The pathogenesis of rheumatoid arthritis may be related to its metabolic dysregulation. Moreover, histological techniques, including genomics, transcriptomics, proteomics and metabolomics, provide powerful tools for comprehensive analysis of molecular changes in biological systems. The present study explores the molecular and metabolic mechanisms of RA, emphasizing the central role of metabolic dysregulation in the RA disease process and highlighting the complexity of metabolic pathways, particularly metabolic remodeling in synovial tissues and its association with cytokine-mediated inflammation. This paper reveals the potential of histological techniques in identifying metabolically relevant therapeutic targets in RA; specifically, we summarize the genetic basis of RA and the dysregulated metabolic pathways, and explore their functional significance in the context of immune cell activation and differentiation. This study demonstrates the critical role of histological techniques in decoding the complex metabolic network of RA and discusses the integration of histological data with other types of biological data.

## 1 Background

Rheumatoid Arthritis (RA) is a chronic inflammatory autoimmune disease affecting approximately 1% of the global population (1). A distinctive feature of RA is the presence of autoantibodies, particularly Rheumatoid Factor (RF) and Anti-Citrullinated Protein Antibodies (ACPA). The pathogenesis of RA involves the development of autoimmunity, localized inflammation, and bone destruction. In RA, metabolic dysregulation reflects the heightened biological energy demands and changes in oxygen and nutrient supply in damaged tissues under a persistent inflammatory state (2). The inflammatory response in the synovial lining is particularly pronounced, with Synovial Tissue Macrophages (STM) and Fibroblast-like Synoviocytes (FLS) exacerbating immune cell infiltration and degradation of cartilage and bone through excessive production of cytokines and enzymes, significantly altering the local metabolic environment (3, 4). Notably, metabolic reprogramming in immune cells is considered a vital source of novel drug targets (5, 6). Therefore, it is crucial to comprehend the metabolic pathways involved in RA and their functional significance.

Over the past decade, the rapid development of omics technologies has greatly enhanced our understanding of the genetic and metabolic mechanisms underlying RA (7). With the advent of the 21st century, the swift progress of high-throughput technologies, Mass Spectrometry (MS) analysis, and single-cell methods has provided powerful tools for in-depth elucidation of the molecular and metabolic mechanisms of RA (8).

This article delves into the molecular and metabolic mechanisms of RA, underscoring the central role of metabolic dysregulation in the disease’s progression. The complexity and functional significance of metabolic pathways in RA are revealed by integrating advances in multi-omics technologies. The article suggests the potential of genomics technologies in identifying metabolism-related therapeutic targets and looks forward to the prospect of multidimensional data fusion for deepening the understanding of RA pathomechanisms.

## 2 RA pathogenesis and metabolic dysregulation

### 2.1 Genetic foundations of RA

The genetic basis of RA exhibits considerable complexity. Research has highlighted the close association between the major histocompatibility complex (MHC) and the genetic predisposition to RA, particularly the polymorphism of human leukocyte antigen (HLA) gene loci, where HLA-DRB1 alleles and their encoded amino acid sequence patterns (shared epitope, SE) play a pivotal role in RA susceptibility (9, 10). The SE is associated with a higher risk of ACPA-positive RA, which in turn is linked to more aggressive RA and cardiovascular complications (11). Recent research has begun to focus on the impact of rare variants on RA susceptibility. Although an enrichment trend for CD2 encoding alleles has been observed in RA, further confirmation is yet to be established (12, 13). Certain HLA-DRB1 alleles in Asians differ structurally from susceptibility alleles in Caucasians, suggesting that genetic risk factors for RA may vary among different populations (14).

### 2.2 Metabolic dysregulation in RA pathogenesis

In the early pathogenic mechanisms of RA, metabolic deviations in synovial cells play a crucial role (2). The typical characteristics of RA include the activation of FLS, characterized by enhanced proliferation, migration, and invasiveness, as well as the activation of STM to produce pro-inflammatory mediators. The pathogenic potential of FLS stems from the immune-regulatory factors, adhesion molecules, and matrix metalloproteinases they express. They are also viewed as “passive responders” in the RA immune response, where their activated state reflects the impact of the pro-inflammatory environment (15). During different stages of RA development, various stimuli such as alterations in glucose and phospholipid metabolism, and unique microenvironmental conditions (like hypoxia and high pressure) prompt the activation and transformation of FLS into an invasive phenotype (16, 17). These stimuli typically activate specific receptors on the cell surface or internally, triggering FLS signaling pathways (18). Activated FLS alter the metabolism of four major macromolecules: proteins, carbohydrates, nucleic acids, and lipids, adopting specific metabolic characteristics to meet their functional requirements.

Studies indicate that stimuli like Tumor Necrosis Factor (TNF) and Platelet-Derived Growth Factor enhance glucose metabolism by promoting mitochondrial respiration and glycolysis (19). Research also reveals an increase in molecules related to lipid metabolism, especially choline (a crucial membrane phospholipid component) in synovial tissue and RA FLS (20, 21). Mitochondria play a pivotal role in cellular metabolism and immune responses. Owing to the limited repair capacity of mitochondrial DNA (mtDNA) and the propensity of oxidative phosphorylation to generate reactive oxygen species (ROS), mtDNA exhibits heightened sensitivity to mutations. Mitochondria not only integrate multiple metabolic pathways to produce intermediates for steroids, lipids, and heme, but also contribute to thermogenesis (22). Under hypoxic conditions, mitochondria generate high levels of ROS, leading to the release of damage-associated molecular patterns (DAMPs) (23), including mtDNA, ATP, and N-formyl peptides, playing a crucial role in non-infectious inflammation (24). Growth factors and cytokines related to RA and associated inflammation, such as TNF, IL-17, and PDGF, induce alterations in FLS mitochondrial metabolism, resulting in excessive ROS production, imbalances in ATP and Ca2+ generated by low-level oxidative phosphorylation, upregulation of nitrogenous compound production, extrinsic cell death, and opening of permeability transition pores (25, 26).

In the pathogenesis of RA, CD4+ T lymphocytes assume a crucial immunoregulatory role. In these cells, a suppression of oxidative phosphorylation and glycolysis leads to a reduction in ATP production, while the primary energy acquisition shifts towards glutaminolysis (27, 28). Furthermore, glucose is diverted from glycolysis to the pentose phosphate pathway, facilitating the accumulation of NADPH and resulting in cell cycle dysregulation and uncontrolled proliferation of T lymphocytes (29). Studies also indicate that B lymphocytes in the RA synovium can be activated by CTLA (30, 31). Additionally, an increase in lactate secretion by macrophages and monocytes not only upregulates the expression of pro-inflammatory cytokines IL-23 and IL-6, thereby promoting the proliferation of Th17 lymphocytes (a principal immune cell group in RA) (32), but also inhibits the migration of CD8+ and CD4+ T lymphocytes, causing their retention at the site of inflammation (33).

**Figure 1:**
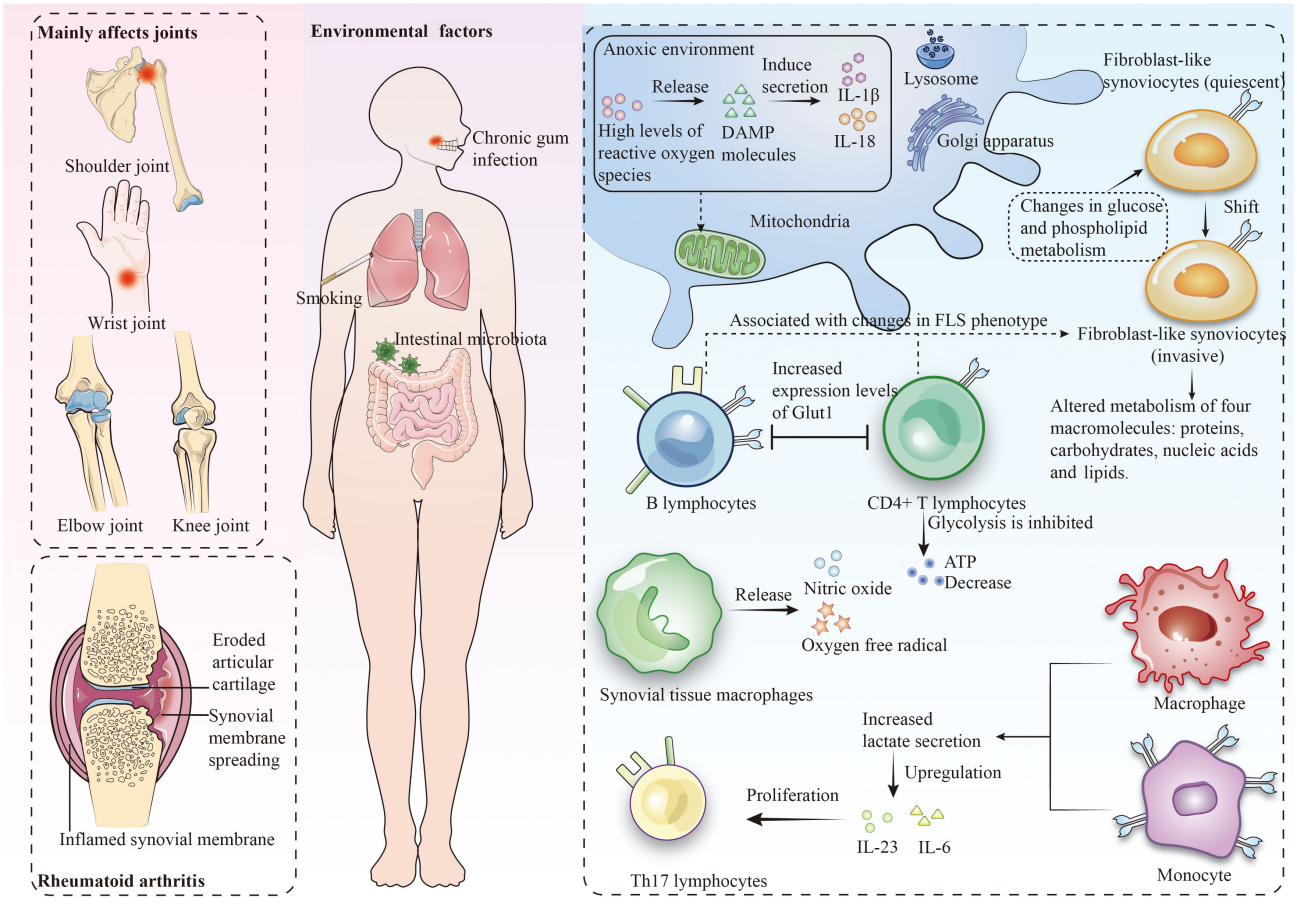
Rheumatoid arthritis predominantly affects the joints and leads to metabolic dysregulation within the context of the immune cell milieu.

In RA joint tissues, macrophages are the most abundant cell type (34). Increasing research emphasizes the significant roles of glycolysis, oxidative phosphorylation, mitochondrial metabolism, and glucose consumption in the differentiation and activation of various macrophage subtypes, such as pro-resolving and pro-inflammatory ones. Under M1 polarization conditions, macrophages are induced by interferon-gamma γ and lipopolysaccharide to adopt a pro-inflammatory state, consequently inhibiting the tricarboxylic acid cycle and related mitochondrial oxidative phosphorylation, and promoting high-level expression and efficient uptake of glucose via Glut1. Furthermore, glucose facilitates the production of substantial amounts of lactate through aerobic glycolysis (35). Peripheral blood macrophages in RA patients exhibit increased levels of glycolysis and oxygen consumption (36). Studies have identified that the HIF-1α factor, active under hypoxic conditions, promotes the transcription of genes for glycolytic enzymes (26). Lactate causes the macrophage microenvironment to become acidic, inducing changes in the glycolytic enzyme pyruvate kinase, which then permeates into the nucleus to activate the STAT3 gene, leading to the production of IL-6 and IL-1β by macrophages (34). Additionally, joint destruction in RA also involves upregulation of cathepsin K protease expression in macrophages (**Figure 1**) (37).

## 3 Applications of omics technologies in the study of metabolic alterations in RA

### 3.1 Genomics

Early studies in genomics primarily focused on determining DNA sequences, involving the analysis of the arrangement of nucleotides in specific DNA segments. With technological advancements, the field has rapidly evolved to a more practical level, encompassing the expression profiles and functional studies of genes and proteins (38). The application of Genome-Wide Association Studies (GWAS) and next-generation sequencing technologies has facilitated the discovery of novel genomic variations. GWAS research has revealed over 100 genetic loci associated with the severity/risk of RA (39). Most other discovered genetic variations are related to guanylation, the immune system, inflammation, and cytokine-related genes, many of which are known therapeutic targets (40). The discovery of these genes not only deepens our understanding of the pathomechanisms of RA, but also opens up new possibilities for personalized treatment (41).

FLS occupy a crucial position in the metabolic processes of RA. Researchers have investigated the potential mechanisms of action of Wantong Jingu Tablet, a prospective effective drug for RA, in FLS (42). Utilizing high-throughput sequencing technology and bioinformatics analysis to screen target genes, the study discovered that the drug potentially promotes apoptosis and inhibits cell proliferation in FLS by suppressing the expression of THOC1, SMC3, STAG2, and BUB1, suggesting that these genes might serve as potential targets for RA treatment. Moreover, the application of whole-genome analysis provides a deeper understanding of the metabolic mechanisms of RA and aids in identifying other key genes and epigenetic biomarkers. DNA microarray technologies now cover all known genes (more than 35,000) and are capable of measuring very small amounts of mRNA molecules within cells, with a lower limit of detection of approximately 10 mRNA copies per cell (43, 44). These technologies are able to measure mRNA molecules over a wide linear range, providing rich data on genetic information in autoimmune diseases. In addition, intra- and inter-platform consistency of genomics technologies has been significantly improved through appropriate gene filtering and probe sequence standardization and optimization (45, 46). Despite significant technological advances, low-abundance genes (e.g., certain cytokines and transcription factors) may be missed or unreliably detected (44). Different probe sequences are used by different technology platforms, leading to differences in the binding characteristics of target genes and thus inconsistent gene expression data.

### 3.2 Metabolomics

Metabolomics is the scientific study of all small molecules or metabolites in biological samples, revealing the functional state of cells and organisms through the analysis of molecular changes. The application of metabolomics not only reveals the responses of cells and organisms in various disease states, growth stages, or under environmental stimuli, but also provides unique insights into biological systems. This aids in disease diagnosis and the discovery of new therapeutic targets.

Researchers utilizing MS analysis of RA synovial tissues have been able to identify specific citrullination sites on fibrinogen (47). However, further research is required on the immunoregulatory role of citrullinated molecules in RA, as these molecules have the potential to serve as novel biomarkers or even as potential therapeutic targets for RA. Utilizing ultra-high performance liquid chromatography-quadrupole-time of flight mass spectrometry, researchers have demonstrated that the levels of tryptophan metabolites in RA synovial fluid are lower compared to those in patients with osteoarthritis. Concurrently, the β-oxidation pathways of cholesterol esters, taurine, and linoleic, oleic, and sphingolipids are more intensely activated in RA patients (48). Another study using the same metabolomics technique found significant changes in RA plasma metabolites and pathways, such as amino acid and lipid metabolism (49). Comparative studies based on metabolomics technology have shown distinct metabolic pathway alterations between newly onset RA (NORA) and chronic RA (CRA) patients (50). These changes are significantly reflected in the dysregulation of histidine, glycerophospholipid metabolism, and the metabolism of serine, glycine, and threonine. Further research revealed significant disturbances in the phenylalanine metabolism pathway in both CRA and NORA patients. Metabolomics pathway analysis revealed the mechanism of action of Aconitum carmichaeli with Ampelopsis japonica in treating RA, identifying three key metabolic pathways: glyceride metabolism, galactose metabolism, and phosphatidylinositol metabolism (51). These significant changes that occur in plasma metabolites and metabolic pathways can help to identify biomarkers of RA, which in turn can lead to the development of new diagnostic tools and therapeutic strategies (**Table 1**). However, metabolomics faces some challenges such as difficulty in identifying many compounds, lack of comprehensive enzyme kinetic data, and complex data processing (55).

**Table 1:** Metabolic pathways of RA revealed by different omics technologies.

Omics technologies	Category	Metabolic pathways	Reference
Genomics	Genome-wide association study	Identification of novel genomic variations potentially impacting RA metabolism.	(39)
	Next-generation sequencing		
Metabolomics	Untargeted metabolomics analysis	Reduction in ornithine, branched-chain amino acids, and aromatic amino acids synthesis.	(52)
	Ultra-high performance liquid chromatography quadrupole time-of-flight mass spectrometry	Enhanced β-oxidation pathways of cholesterol esters, taurine, linolenic acid, arachidonic acid, and sphingolipids.	(48)
		Significant alterations in amino acid metabolism and lipid metabolism.	(49)
	–	Significant disruption in phenylalanine metabolic pathways.	(50)
	Mass spectrometry	Tryptophan metabolite levels are associated with rheumatoid factors, inflammatory markers, and anti-cyclic citrullinated peptide antibody levels.	(48)
Transcriptomics	RNA sequencing	The key determinant of Th phenotype differentiation is polyamine metabolism.	(53)
		Dysregulation of glycerophospholipid metabolism.	(50)
Proteomics	Protein-protein interaction network analysis	Interaction between ACHE and LYPLA1, LCAT proteins leading to alterations in metabolic products of glycerophospholipid metabolism pathway in RA.	(50)
	–	Deletion of target genes can lead to positive or negative changes in metabolic reactions controlled by upregulated or downregulated genes in the disease, emphasizing the importance of Th1 and Th2 phenotypes in the metabolic network.	(54)

ACHE, Acetylcholinesterase, LYPLA1, Phospholipase A1 Member 1; LCAT, Lecithin-Cholesterol Acyltransferase.

### 3.3 Transcriptomics

Transcriptomic research, utilizing real-time PCR and advanced microarray technologies, has become a routine scientific inquiry method. RNA sequencing (RNA-seq), capable of covering a broader range of RNA types and providing richer information, occupies a significant position in transcriptomic studies (56). However, advancements are still needed in identifying reliable gene expression patterns (57). Transcriptomics also provides a picture of the dynamics of gene expression in patients before and after different treatments, which allows for the assessment of treatment efficacy. For example, before and after anti-IL-6 and TNFα treatments, there are significant changes in patients’ gene expression patterns, which can help predict treatment response (58). However, transcriptomics technologies are complex for data interpretation and require advanced statistical and bioinformatics skills to manage and interpret large amounts of data. And the relatively high cost of single-cell RNA-seq may limit its use in routine clinical practice (59).

In immunological research, single-cell RNA-seq has garnered widespread attention (60, 61), with researchers like Cheung et al. focusing on its application in rheumatologic diseases (62). Single-cell RNA-seq analysis of RA synovium has identified three distinct fibroblast subgroups (two sub lining and one lining), each with unique transcriptional profiles (63, 64). The inflammatory phenotype of synovial fibroblasts is associated with changes in glucose metabolism (65), and these subpopulations may be potential drug targets independent of the immune system. Combining the network topology of human metabolic profiles with single-cell RNA-seq and applying a metabolic model in the form of flux balance analysis (FBA), the researchers found that a key determinant of Th phenotypic differentiation is polyamine metabolism (53). In another study, RNA-seq was used to analyze gene expression profiles in NORA and CRA compared to control groups. The comparative study between NORA and the control group revealed significant enrichment of nine gene sets in the control group, covering key biological processes such as glycerophospholipid metabolism, calcium signaling pathway, and neuroactive ligand-receptor interaction. Meanwhile, in the CRA versus control group study, 16 gene sets were found to be enriched in CRA patients (such as the citric acid cycle), compared to only three in the control group. KEGG enrichment analysis of differentially expressed genes between CRA and NORA revealed three significantly dysregulated metabolic pathways: glycerophospholipid metabolism, glycerophospholipid biosynthesis - glycerol series, and proximal tubule bicarbonate reclamation, providing new insights into the complex metabolic network of RA (50).

Furthermore, most transcriptomic studies in rheumatology have utilized T-cell receptor (TCR) sequencing. Exploring TCR diversity in RA using RNA-seq technology for the TCR β-chain, researchers have found significant overlaps of dominant TCR clones within affected joints and synovial regions, suggesting the presence of targetable lymphocytes with therapeutic implications in RA (66). The interactions between lymphocytes (T cells, B cells, etc.), inflammatory cells, synovial cells, and cytokines are part of the RA metabolic mechanism, involving immune responses and inflammatory regulation.

### 3.4 Proteomics

The essence of proteomics lies in the comprehensive analysis of proteins in tissue samples or biological fluids, including their expression, function, structure, chemical modifications, and interactions. Research methods are diverse and efficient, covering microarray-based technologies as well as the latest single-cell and high-sensitivity protein analysis methods (67, 68). Proteomics in RA is utilized to identify peptide mediators and key proteins (69), and also demonstrates unique value in the detection and quantification of cytokines. These technologies not only facilitate early diagnosis of RA but also provide potential biomarkers for monitoring disease progression and treatment response (70).

Proteomic-protein interaction network analysis in RA has identified three core genes: Fibronectin 1 (FN1), Acetylcholinesterase (ACHE), and Aquaporin 1 (AQP1) (50). ACHE is associated with AQP1, Lysophospholipase I (LYPLA1), and FN1, while tyrosine aminotransferase interacts with glucokinase regulatory protein, and LYPLA1 is linked with lecithin-cholesterol acyltransferase (LCAT). Additionally, an upregulation of ACHE in the glycerophospholipid metabolism pathway and different downstream metabolites of LYPLA1 protein and LCAT (such as glycerophosphocholine) were observed. Studies indicate that interactions between ACHE and LYPLA1, LCAT proteins lead to changes in the metabolic products of the glycerophospholipid pathway in RA. In another study, using proteomic and transcriptomic data, a genome-scale metabolic model based on T-cell phenotypes was established, and specific metabolic genes that could serve as therapeutic targets in autoimmune diseases like RA were identified through FBA (54). The study suggests that the absence of target genes would lead to positive or negative changes in metabolic reactions controlled by genes that are downregulated or upregulated in the disease, highlighting the importance of Th1 and Th2 phenotypes in the metabolic network. Th1 cells exhibit a stronger glycolytic flux compared to Th2 cells, suggesting that glycolysis in Th1 cells could be a predictive target for RA. Researchers believe that metabolic dysregulation driving the transition of T-cell phenotypes may induce the development of autoimmune diseases. Furthermore, IL-6 inhibits Transforming Growth Factor β, impairing Treg phenotype and thus reducing the inhibition of adaptive T-cell responses, promoting the formation of Th17 cells, which are associated with the incidence and severity of RA (71, 72).

Protein microarray technology is capable of detecting up to 10 cell equivalents. Combined with the high quality accuracy of mass spectrometry (<10 ppm), it is capable of identifying any protein in the sample (73). 2D gel electrophoresis combined with mass spectrometry allows for the efficient separation and identification of specific proteins in a sample (74). Despite the high resolution, the number of proteins that can be identified by current techniques is typically less than 10,000, whereas the number of known proteins is approximately one million. Proteomics techniques are poorly reproducible across experiments and lack reliable methods for quantitative analysis.

## 4 Discussion

This study delves into the metabolic pathways of RA, highlighting the significant contributions of omics technologies in this domain. Studies of RA metabolic pathways not only reveal pathological mechanisms, but are also critical for identifying new diagnostic markers and therapeutic targets. RA treatment strategies necessitate the integration of multifaceted data to enhance treatment precision. Omics data provide molecular level insights, while the integration with imaging and clinical data can provide biological relevance for these molecular findings. Specific metabolic changes may be associated with the degree of joint inflammation or bone destruction observed on imaging. Furthermore, a significant challenge in employing omics technologies in RA research lies in managing and interpreting complex biological information within large-scale datasets. Therefore, it is imperative to incorporate systems biology strategies and leverage advanced modeling tools to address the variability, complexity, and non-linear features of biological interactions (75). Modelling RA biology is also challenging and requires careful analysis based on the type of experimental data available using a variety of mathematical and computational tools (76).

## Author contributions

XG: Data curation, Writing – original draft. LS: Data curation, Writing – original draft. JH: Writing – original draft. JL: Writing – original draft. QW: Writing – original draft. XL: Conceptualization, Writing – original draft, Writing – review & editing. GY: Conceptualization, Writing – original draft, Writing – review & editing. HC: Conceptualization, Data curation, Writing – original draft, Writing – review & editing.

## References

[1] Scott DL Wolfe F Huizinga TW. Rheumatoid arthritis. Lancet. (2010) 376:1094–108. doi: 10.1016/S0140-6736(10)60826-4 20870100

[2] Falconer J Murphy AN Young SP Clark AR Tiziani S Guma M. Review: synovial cell metabolism and chronic inflammation in rheumatoid arthritis. Arthritis Rheumatol. (2018) 70:984–99. doi: 10.1002/art.40504 PMC6019623 29579371

[3] Sanchez-Lopez E Cheng A Guma M. Can metabolic pathways be therapeutic targets in rheumatoid arthritis? J Clin Med. (2019) 8:753. doi: 10.3390/jcm8050753 31137815 PMC6572063

[4] MaChado CRL Dias FF Resende GG Oliveira PG Xavier RM Andrade MVM. Morphofunctional analysis of fibroblast-like synoviocytes in human rheumatoid arthritis and mouse collagen-induced arthritis. Adv Rheumatol. (2023) 63:1. doi: 10.1186/s42358-022-00281-0 36597166

[5] O'Neill LA Kishton RJ Rathmell J. A guide to immunometabolism for immunologists. Nat Rev Immunol. (2016) 16:553–65. doi: 10.1038/nri.2016.70 PMC5001910 27396447

[6] Cai WW Yu Y Zong SY Wei F. Metabolic reprogramming as a key regulator in the pathogenesis of rheumatoid arthritis. Inflammation Res. (2020) 69:1087–101. doi: 10.1007/s00011-020-01391-5 32797249

[7] Kim HY Kim HR Lee SH. Advances in systems biology approaches for autoimmune diseases. Immune Netw. (2014) 14:73–80. doi: 10.4110/in.2014.14.2.73 24851096 PMC4022781

[8] Cassotta M Forbes-Hernandez TY Cianciosi D Elexpuru Zabaleta M Sumalla Cano S Dominguez I. Nutrition and rheumatoid arthritis in the 'Omics' Era. Nutrients. (2021) 13:763. doi: 10.3390/nu13030763 33652915 PMC7996781

[9] Raychaudhuri S. Recent advances in the genetics of rheumatoid arthritis. Curr Opin Rheumatol. (2010) 22:109–18. doi: 10.1097/BOR.0b013e328336474d PMC3121048 20075733

[10] Ting YT Petersen J Ramarathinam SH Scally SW Loh KL Thomas R. The interplay between citrullination and HLA-DRB1 polymorphism in shaping peptide binding hierarchies in rheumatoid arthritis. J Biol Chem. (2018) 293:3236–51. doi: 10.1074/jbc.RA117.001013 PMC5836122 29317506

[11] Murphy D Mattey D Hutchinson D. Anti-citrullinated protein antibody positive rheumatoid arthritis is primarily determined by rheumatoid factor titre and the shared epitope rather than smoking per se. PloS One. (2017) 12:e0180655. doi: 10.1371/journal.pone.0180655 28708862 PMC5510819

[12] Diogo D Kurreeman F Stahl EA Liao KP Gupta N Greenberg JD. Rare, low-frequency, and common variants in the protein-coding sequence of biological candidate genes from GWASs contribute to risk of rheumatoid arthritis. Am J Hum Genet. (2013) 92:15–27. doi: 10.1016/j.ajhg.2012.11.012 23261300 PMC3542467

[13] Okada Y Eyre S Suzuki A Kochi Y Yamamoto K. Genetics of rheumatoid arthritis: 2018 status. Ann Rheum Dis. (2019) 78:446–53. doi: 10.1136/annrheumdis-2018-213678 30530827

[14] Mackie SL Taylor JC Martin SG YEAR Consortium UKRAG Consortium Wordsworth P. A spectrum of susceptibility to rheumatoid arthritis within HLA-DRB1: stratification by autoantibody status in a large UK population. Genes Immun. (2012) 13:120–8. doi: 10.1038/gene.2011.60 21881596

[15] Bottini N Firestein GS. Duality of fibroblast-like synoviocytes in RA: passive responders and imprinted aggressors. Nat Rev Rheumatol. (2013) 9:24–33. doi: 10.1038/nrrheum.2012.190 23147896 PMC3970924

[16] Bustamante MF Garcia-Carbonell R Whisenant KD Guma M. Fibroblast-like synoviocyte metabolism in the pathogenesis of rheumatoid arthritis. Arthritis Res Ther. (2017) 19:110. doi: 10.1186/s13075-017-1303-3 28569176 PMC5452638

[17] Ng CT Biniecka M Kennedy A McCormick J Fitzgerald O Bresnihan B. Synovial tissue hypoxia and inflammation in vivo. Ann Rheum Dis. (2010) 69:1389–95. doi: 10.1136/ard.2009.119776 PMC2946116 20439288

[18] Hammaker D Sweeney S Firestein GS. Signal transduction networks in rheumatoid arthritis. Ann Rheum Dis. (2003) 62 Suppl 2:ii86–9. doi: 10.1136/ard.62.suppl_2.ii86 PMC1766749 14532158

[19] Garcia-Carbonell R Divakaruni AS Lodi A Vicente-Suarez I Saha A Cheroutre H. Critical role of glucose metabolism in rheumatoid arthritis fibroblast-like synoviocytes. Arthritis Rheumatol. (2016) 68:1614–26. doi: 10.1002/art.39608 PMC4963240 26815411

[20] Ahn JK Kim S Hwang J Kim J Kim KH Cha HS. GC/TOF-MS-based metabolomic profiling in cultured fibroblast-like synoviocytes from rheumatoid arthritis. Joint Bone Spine. (2016) 83:707–13. doi: 10.1016/j.jbspin.2015.11.009 27133762

[21] Volchenkov R Dung Cao M Elgstøen KB Goll GL Eikvar K Bjørneboe O. Metabolic profiling of synovial tissue shows altered glucose and choline metabolism in rheumatoid arthritis samples. Scand J Rheumatol. (2017) 46:160–1. doi: 10.3109/03009742.2016.1164242 27098118

[22] Osellame LD Blacker TS Duchen MR. Cellular and molecular mechanisms of mitochondrial function. Best Pract Res Clin Endocrinol Metab. (2012) 26:711–23. doi: 10.1016/j.beem.2012.05.003 PMC3513836 23168274

[23] Filippin LI Vercelino R Marroni NP Xavier RM. Redox signalling and the inflammatory response in rheumatoid arthritis. Clin Exp Immunol. (2008) 152:415–22. doi: 10.1111/j.1365-2249.2008.03634.x PMC2453196 18422737

[24] Grazioli S Pugin J. Mitochondrial damage-associated molecular patterns: from inflammatory signaling to human diseases. Front Immunol. (2018) 9:832. doi: 10.3389/fimmu.2018.00832 29780380 PMC5946030

[25] Kim EK Kwon JE Lee SY Lee EJ Kim DS Moon SJ. IL-17-mediated mitochondrial dysfunction impairs apoptosis in rheumatoid arthritis synovial fibroblasts through activation of autophagy. Cell Death Dis. (2017) 8:e2565. doi: 10.1038/cddis.2016.490 PMC5386390 28102843

[26] Fearon U Canavan M Biniecka M Veale DJ. Hypoxia, mitochondrial dysfunction and synovial invasiveness in rheumatoid arthritis. Nat Rev Rheumatol. (2016) 12:385–97. doi: 10.1038/nrrheum.2016.69 27225300

[27] Qiu J Wu B Goodman SB Berry GJ Goronzy JJ Weyand CM. Metabolic control of autoimmunity and tissue inflammation in rheumatoid arthritis. Front Immunol. (2021) 12:652771. doi: 10.3389/fimmu.2021.652771 33868292 PMC8050350

[28] Araujo L Khim P Mkhikian H Mortales CL Demetriou M. Glycolysis and glutaminolysis cooperatively control T cell function by limiting metabolite supply to N-glycosylation. Elife. (2017) 6:e21330. doi: 10.7554/eLife.21330 28059703 PMC5257256

[29] Yang Z Shen Y Oishi H Matteson EL Tian L Goronzy JJ. Restoring oxidant signaling suppresses proarthritogenic T cell effector functions in rheumatoid arthritis. Sci Transl Med. (2016) 8:331ra38. doi: 10.1126/scitranslmed.aad7151 PMC5074090 27009267

[30] Souto-Carneiro MM Klika KD Abreu MT Meyer AP Saffrich R Sandhoff R. Effect of increased lactate dehydrogenase A activity and aerobic glycolysis on the proinflammatory profile of autoimmune CD8+ T cells in rheumatoid arthritis. Arthritis Rheumatol. (2020) 72:2050–64. doi: 10.1002/art.41420 32602217

[31] Silverman GJ Carson DA. Roles of B cells in rheumatoid arthritis . Arthritis Res Ther. (2003) 5 Suppl 4:S1–6. doi: 10.1186/ar1010 PMC2833442 15180890

[32] Shime H Yabu M Akazawa T Kodama K Matsumoto M Seya T. Tumor-secreted lactic acid promotes IL-23/IL-17 proinflammatory pathway. J Immunol. (2008) 180:7175–83. doi: 10.4049/jimmunol.180.11.7175 18490716

[33] Haas R Smith J Rocher-Ros V Nadkarni S Montero-Melendez T D'Acquisto F. Lactate regulates metabolic and pro-inflammatory circuits in control of T cell migration and effector functions. PloS Biol. (2015) 13:e1002202. doi: 10.1371/journal.pbio.1002202 26181372 PMC4504715

[34] Weyand CM Zeisbrich M Goronzy JJ. Metabolic signatures of T-cells and macrophages in rheumatoid arthritis. Curr Opin Immunol. (2017) 46:112–20. doi: 10.1016/j.coi.2017.04.010 PMC5554742 28538163

[35] Corcoran SE O'Neill LA. HIF1α and metabolic reprogramming in inflammation. J Clin Invest. (2016) 126:3699–707. doi: 10.1172/JCI84431 PMC5096812 27571407

[36] Zeisbrich M Yanes RE Zhang H Watanabe R Li Y Brosig L. Hypermetabolic macrophages in rheumatoid arthritis and coronary artery disease due to glycogen synthase kinase 3b inactivation. Ann Rheum Dis. (2018) 77:1053–62. doi: 10.1136/annrheumdis-2017-212647 PMC6589337 29431119

[37] Yamashita T Assimes TL Goronzy JJ Weyand CM. Effect of a cathepsin K inhibitor on arthritis and bone mineral density in ovariectomized rats with collagen-induced arthritis. Bone Rep. (2018) 9:1–10. doi: 10.1016/j.bonr.2018.05.006 29992179 PMC6034140

[38] Del Giacco L Cattaneo C. Introduction to genomics. Methods Mol Biol. (2012) 823:79–88. doi: 10.1007/978-1-60327-216-2_6 22081340

[39] Okada Y Wu D Trynka G Raj T Terao C Ikari K. Genetics of rheumatoid arthritis contributes to biology and drug discovery. Nature. (2014) 506:376–81. doi: 10.1038/nature12873 PMC3944098 24390342

[40] Korczowska I. Rheumatoid arthritis susceptibility genes: An overview. World J Orthop. (2014) 5:544–9. doi: 10.5312/wjo.v5.i4.544 PMC4133460 25232530

[41] Goulielmos GN Zervou MI Myrthianou E Burska A Niewold TB Ponchel F. Genetic data: The new challenge of personalized medicine, insights for rheumatoid arthritis patients. Gene. (2016) 583:90–101. doi: 10.1016/j.gene.2016.02.004 26869316

[42] Li Z Qi F Li F. Identification of drug targets and potential molecular mechanisms for Wantong Jingu Tablet extract in treatment of rheumatoid arthritis: bioinformatics analysis of fibroblast-like synoviocytes. Chin Med. (2020) 15:59. doi: 10.1186/s13020-020-00339-5 32518584 PMC7275334

[43] Lockhart DJ Dong H Byrne MC Follettie MT Gallo MV Chee MS. Expression monitoring by hybridization to high-density oligonucleotide arrays. Nat Biotechnol. (1996) 14:1675–80. doi: 10.1038/nbt1296-1675 9634850

[44] Draghici S Khatri P Eklund AC Szallasi Z. Reliability and reproducibility issues in DNA microarray measurements. Trends Genet. (2006) 22:101–9. doi: 10.1016/j.tig.2005.12.005 PMC2386979 16380191

[45] Reverter A McWilliam SM Barris W Dalrymple BP. A rapid method for computationally inferring transcriptome coverage and microarray sensitivity. Bioinformatics. (2005) 21:80–9. doi: 10.1093/bioinformatics/bth472 15308544

[46] MAQC Consortium Shi L Reid LH Jones WD Shippy R Warrington JA. The MicroArray Quality Control (MAQC) project shows inter- and intraplatform reproducibility of gene expression measurements. Nat Biotechnol. (2006) 24:1151–61. doi: 10.1038/nbt1239 PMC3272078 16964229

[47] Hermansson M Artemenko K Ossipova E Eriksson H Lengqvist J Makrygiannakis D. MS analysis of rheumatoid arthritic synovial tissue identifies specific citrullination sites on fibrinogen. Proteomics Clin Appl. (2010) 4:511–8. doi: 10.1002/prca.200900088 21137068

[48] Kang KY Lee SH Jung SM Park SH Jung BH Ju JH. Downregulation of tryptophan-related metabolomic profile in rheumatoid arthritis synovial fluid. J Rheumatol. (2015) 42:2003–11. doi: 10.3899/jrheum.141505 26329338

[49] Zhu J Wang T Lin Y Xiong M Chen J Jian C. The change of plasma metabolic profile and gut microbiome dysbiosis in patients with rheumatoid arthritis. Front Microbiol. (2022) 13:931431. doi: 10.3389/fmicb.2022.931431 36329847 PMC9623673

[50] Jian C Wei L Wu T Li S Wang T Chen J. Comprehensive multi-omics analysis reveals the core role of glycerophospholipid metabolism in rheumatoid arthritis development. Arthritis Res Ther. (2023) 25:246. doi: 10.1186/s13075-023-03208-2 38102690 PMC10722724

[51] Jin H Ma N Li X Kang M Guo M Song L. Application of GC/MS-Based Metabonomic Profiling in Studying the Therapeutic Effects of Aconitum carmichaeli with Ampelopsis japonica Extract on Collagen-Induced Arthritis in Rats. Molecules. (2019) 24:1934. doi: 10.3390/molecules24101934 31137469 PMC6571615

[52] Yu D Du J Pu X Zheng L Chen S Wang N. The gut microbiome and metabolites are altered and interrelated in patients with rheumatoid arthritis. Front Cell Infect Microbiol. (2021) 11:763507. doi: 10.3389/fcimb.2021.763507 35145919 PMC8821809

[53] Wagner A Wang C Fessler J DeTomaso D Avila-Pacheco J Kaminski J. Metabolic modeling of single Th17 cells reveals regulators of autoimmunity. Cell. (2021) 184:4168–4185.e21. doi: 10.1016/j.cell.2021.05.045 34216539 PMC8621950

[54] Puniya BL Amin R Lichter B Moore R Ciurej A Bennett SJ. Integrative computational approach identifies drug targets in CD4(+) T-cell-mediated immune disorders. NPJ Syst Biol Appl. (2021) 7:4. doi: 10.1038/s41540-020-00165-3 33483502 PMC7822845

[55] Downs DM Bazurto JV Gupta A Fonseca LL Voit EO. The three-legged stool of understanding metabolism: integrating metabolomics with biochemical genetics and computational modeling. AIMS Microbiol. (2018) 4:289–303. doi: 10.3934/microbiol.2018.2.289 31294216 PMC6604926

[56] Zhao S Fung-Leung WP Bittner A Ngo K Liu X. Comparison of RNA-Seq and microarray in transcriptome profiling of activated T cells. PloS One. (2014) 9:e78644. doi: 10.1371/journal.pone.0078644 24454679 PMC3894192

[57] Byron SA Van Keuren-Jensen KR Engelthaler DM Carpten JD Craig DW. Translating RNA sequencing into clinical diagnostics: opportunities and challenges. Nat Rev Genet. (2016) 17:257–71. doi: 10.1038/nrg.2016.10 PMC7097555 26996076

[58] Sumitomo S Nagafuchi Y Tsuchida Y Tsuchiya H Ota M Ishigaki K. Transcriptome analysis of peripheral blood from patients with rheumatoid arthritis: a systematic review. Inflammation Regener. (2018) 38:21. doi: 10.1186/s41232-018-0078-5 PMC6217768 30410636

[59] Zhang F Wei K Slowikowski K Fonseka CY Rao DA Kelly S. Defining inflammatory cell states in rheumatoid arthritis joint synovial tissues by integrating single-cell transcriptomics and mass cytometry. Nat Immunol. (2019) 20:928–42. doi: 10.1038/s41590-019-0378-1 PMC6602051 31061532

[60] Giladi A Amit I. Single-cell genomics: A stepping stone for future immunology discoveries. Cell. (2018) 172:14–21. doi: 10.1016/j.cell.2017.11.011 29328909

[61] Landhuis E. S ingle-cell approaches to immune profiling. Nature. (2018) 557:595–7. doi: 10.1038/d41586-018-05214-w 29789748

[62] Cheung P Khatri P Utz PJ Kuo AJ. Single-cell technologies - studying rheumatic diseases one cell at a time. Nat Rev Rheumatol. (2019) 15:340–54. doi: 10.1038/s41584-019-0220-z PMC7051208 31065108

[63] Mizoguchi F Slowikowski K Wei K Marshall JL Rao DA Chang SK. Functionally distinct disease-associated fibroblast subsets in rheumatoid arthritis. Nat Commun. (2018) 9:789. doi: 10.1038/s41467-018-02892-y 29476097 PMC5824882

[64] Stephenson W Donlin LT Butler A Rozo C Bracken B Rashidfarrokhi A. Single-cell RNA-seq of rheumatoid arthritis synovial tissue using low-cost microfluidic instrumentation. Nat Commun. (2018) 9:791. doi: 10.1038/s41467-017-02659-x 29476078 PMC5824814

[65] Masuko K. Glucose as a potential key to fuel inflammation in rheumatoid arthritis. Nutrients. (2022) 14:2349. doi: 10.3390/nu14112349 35684149 PMC9182926

[66] Musters A Klarenbeek PL Doorenspleet ME Balzaretti G Esveldt REE van Schaik BDC. In Rheumatoid Arthritis, Synovitis at Different Inflammatory Sites Is Dominated by Shared but Patient-Specific T Cell Clones. J Immunol. (2018) 201:417–22. doi: 10.4049/jimmunol.1800421 29891556

[67] Parker SJ Raedschelders K Van Eyk JE. Emerging proteomic technologies for elucidating context-dependent cellular signaling events: A big challenge of tiny proportions. Proteomics. (2015) 15:1486–502. doi: 10.1002/pmic.201400448 PMC4743877 25545106

[68] Levy E Slavov N. Single cell protein analysis for systems biology. Essays Biochem. (2018) 62:595–605. doi: 10.1042/EBC20180014 30072488 PMC6204083

[69] Mahendran SM Keystone EC Krawetz RJ Liang K Diamandis EP Chandran V. Elucidating the endogenous synovial fluid proteome and peptidome of inflammatory arthritis using label-free mass spectrometry. Clin Proteomics. (2019) 16:23. doi: 10.1186/s12014-019-9243-3 31160890 PMC6542032

[70] Burska A Boissinot M Ponchel F. Cytokines as biomarkers in rheumatoid arthritis. Mediators Inflammation. (2014) 2014:545493. doi: 10.1155/2014/545493 PMC3964841 24733962

[71] Kimura A Kishimoto T. IL-6: regulator of Treg/Th17 balance. Eur J Immunol. (2010) 40:1830–5. doi: 10.1002/eji.201040391 20583029

[72] Vyas SP Hansda AK Goswami R. Rheumatoid arthritis: 'melting pot' of T helper subsets. Int Rev Immunol. (2019) 38:212–31. doi: 10.1080/08830185.2019.1621865 31155981

[73] Kunz M Ibrahim SM. Cytokines and cytokine profiles in human autoimmune diseases and animal models of autoimmunity. Mediators Inflammation. (2009) 2009:979258. doi: 10.1155/2009/979258 PMC2768824 19884985

[74] Song X Lin Q. Genomics, transcriptomics and proteomics to elucidate the pathogenesis of rheumatoid arthritis. Rheumatol Int. (2017) 37:1257–65. doi: 10.1007/s00296-017-3732-3 28493174

[75] Purohit V Wagner A Yosef N Kuchroo VK. Systems-based approaches to study immunometabolism. Cell Mol Immunol. (2022) 19:409–20. doi: 10.1038/s41423-021-00783-9 PMC8891302 35121805

[76] Robert PA Kunze-Schumacher H Greiff V Krueger A. Modeling the dynamics of T-cell development in the thymus. Entropy (Basel). (2021) 23:437. doi: 10.3390/e23040437 33918050 PMC8069328

